# Dependence of brain DTI maps of fractional anisotropy and mean diffusivity on the number of diffusion weighting directions

**DOI:** 10.1120/jacmp.v11i1.2927

**Published:** 2009-12-23

**Authors:** Marco Giannelli, Mirco Cosottini, Maria Chiara Michelassi, Guido Lazzarotti, Gina Belmonte, Carlo Bartolozzi, Mauro Lazzeri

**Affiliations:** ^1^ Unit of Medical Physics Azienda Ospedaliero‐Universitaria Pisana Pisa Italy; ^2^ Department of Neuroscience University of Pisa Pisa Italy; ^3^ Unit of Neuroradiology Azienda Ospedaliero‐Universitaria Pisana Pisa Italy; ^4^ Department of Physics University of Pisa Pisa Italy; ^5^ Department of Oncology Transplants and Advanced Technologies in Medicine University of Pisa Pisa Italy

**Keywords:** magnetic resonance imaging, diffusion tensor imaging, diffusion, fractional anisotropy, mean diffusivity

## Abstract

The rotational variance dependence of diffusion tensor imaging (DTI) derived parameters on the number of diffusion weighting directions (N) has been investigated by several Monte Carlo simulation studies. However, the dependence of fractional anisotropy (FA) and mean diffusivity (MD) maps on N, in terms of accuracy and contrast between different anatomical structures, has not been assessed in detail. This experimental study further investigated *in vivo* the effect of the number of diffusion weighting directions on DTI maps of FA and MD. Human brain FA and MD maps of six healthy subjects were acquired at 1.5T with varying N (6, 11, 19, 27, 55). Then, FA and MD mean values in high $\left({\text{FA}}_{H},\;{\text{MD}}_{H}\right)$ and low $\left({\text{FA}}_{L},\;{\text{MD}}_{L}\right)$ anisotropy segmented brain regions were measured. Moreover, the contrast‐to‐signal variance ratio $\left({\text{CVR}}_{\text{FA}},\;{\text{CVR}}_{\text{MD}}\right)$ between the main white matter and the surrounding regions was calculated. Analysis of variance showed that ${\text{FA}}_{L},\;{\text{FA}}_{H}$ and ${\text{CVR}}_{\text{FA}}$ significantly (p<0.05) depend on N. In particular, FAL decreased (6%–11%) with N, whereas ${\text{FA}}_{H}$ (1.6%–2.5%) and ${\text{CVR}}_{\text{FA}}$ (4%–6.5%) increased with $N.\;{\text{MD}}_{L},\;{\text{MD}}_{H}$ and ${\text{CVR}}_{\text{MD}}$ did not significantly (p>0.05) depend on N. Unlike MD values, FA values significantly vary with N. It is noteworthy that the observed variation is opposite in low and high anisotropic regions. In clinical studies, the effect of N may represent a confounding variable for anisotropy measurements and the employment of DTI acquisition schemes with high N(>20) allows an increased CVR and a better visualization of white matter structures in FA maps.

PACS number: 87.61.Tg, 82.56.Lz

## I. INTRODUCTION

Diffusion‐weighted magnetic resonance imaging (DW‐MRI) is an MRI technique sensitized to diffusive properties of water molecules, which offers the possibility of noninvasively characterizing diffusion *in vivo*. Water diffusion is a three‐dimensional process which can be described by means of the effective symmetric diffusion tensor (D). Diffusion tensor MR imaging (DTI) represents the NMR measurement of diffusion tensor (D) and the analysis and display of information that it contains.^(1)^ DTI provides a useful tool to measure water diffusion and tissues anisotropy.^(2)^

Fractional anisotropy (FA) is a scalar measure that can be extracted from diffusion tensor to display quantitative maps.^(3)^ FA quantifies the amount of anisotropy of water diffusion within tissues, and it is a rotationally invariant index which allows orientation‐independent comparisons of diffusion anisotropy values between different subjects.^(4)^ Mean diffusivity (MD) is a DTI‐derived parameter, rotationally invariant, which quantifies water diffusion within tissues.^(4)^ FA and MD can be used to characterize ultrastructural properties and integrity of brain structures, and have proved to be very useful in studying and revealing early changes in many neurological diseases.^(5)^ In neuroimaging, DTI analyses of FA and MD have shown their potential in various disorders such as multiple sclerosis,^(^ ^6^ ^,^ ^7^ ^)^ amyotrophic lateral sclerosis,^(^ ^8^ ^,^ ^9^ ^)^ cerebral neoplasm,^(10)^ ischemia,^(^ ^11^ ^,^ ^12^ ^)^ epilepsy,^(^ ^13^ ^,^ ^14^ ^)^ human brain development,^(15)^ and Alzheimer's disease.^(16)^

Diffusion tensor can be estimated from at least six diffusion‐weighted images acquired along noncollinear directions and one diffusion unweighted image. To improve the accuracy and precision of diffusion tensor estimation, more than six diffusion‐weighted images can be acquired along six or more noncollinear directions, resulting in an increased total acquisition time. Therefore, there are two options: either to add additional diffusion‐weighting directions or repeat existing diffusion‐weighting directions. However, there are some basic requirements (multiple diffusion‐weighting directions, several relatively thin sections in order to accurately characterize different anatomical structures and cover the entire brain) which make DTI a time‐demanding technique. Since, in a clinical acquisition, the total scan time cannot be too long, it is fundamental and of practical interest to optimize DTI acquisitions at fixed acquisition time. In this regard, theoretic analyses and Monte Carlo simulations have suggested that DTI results seem to depend on the applied acquisition scheme.^(^ ^17^ ^–^ ^21^ ^)^ In particular, acquisition schemes with a high number of diffusion‐weighting directions (N), and that sample the space uniformly, could improve anisotropy measurements and reduce rotational variance due to noise propagation.^(^ ^22^ ^–^ ^24^ ^)^ The rotational variance dependence of DTI derived parameters on the number of diffusion‐weighting directions (N) has been investigated by several simulation studies. However, the dependence of FA and MD maps on N, in terms of accuracy and contrast between different anatomical structures, has not been assessed in detail. Only Poonawalla and Zhou^(25)^ and Ni et al.^(26)^ have reported quantitative human brain DTI data of a multisubject investigation. Poonawalla and Zhou^(25)^ have executed solely ROI‐based FA measurements of the splenium of corpus callosum (very high anisotropy, FA>0.7) and, applying an analytical formalism, have suggested that precision (standard deviation) of FA measurements seems to improve as N increases. Ni et al.^(26)^ have performed in six highly directional white matter structures (FA>0.5) a quantitative analysis that accounts for the DTI indexes’ dependence on acquisition schemes with different N by using a limited number of DTI acquisition protocols (N=6, 21, 31). Landman et al.^(27)^ have reported single‐subject data of a study which has investigated the effects of diffusion‐weighting schemes, with N up to 30, on the reproducibility of measurements of DTI‐derived parameters. It should be noted that no previous study has performed quantitative measurements to assess the effect of N on the contrast between different brain structures in FA and MD maps; Skare et al.^(20)^ and Jones et al.^(28)^ have reported only qualitative observations.

Based on these considerations, and to obtain further insight into the dependence of maps of DTI‐derived parameters on acquisition schemes with different N, we experimentally evaluated in a healthy subjects group the accuracy of both FA and MD values in segmented brain regions of high and low anisotropy by employing N up to 55. Moreover, we quantitatively investigated whether the capability of FA and MD maps to characterize different structures varies with N.

## II. MATERIALS AND METHODS

All acquisitions were performed on a clinical 1.5T MR scanner (Signa Infinity Twinspeed, GE Medical Systems ‐ Milwaukee, WI) with 40 mT/m maximum gradient strength and 150 T/m/s slew rate. A standard quadrature head coil with a diameter of 26 cm was used for RF transmission and reception of the NMR signal.

Diffusion‐weighted images were obtained with a spin echo – echo planar imaging sequence sensitized to water diffusion (DWI‐SE‐EPI) by a strong magnetic field gradient pulse.^(29)^

To reduce intersubject differences, a total of six healthy volunteers (three males, three females) of comparable age (29±4 years) with no history of neurological disease underwent DTI measurements. Written informed consent to participation was obtained from all subjects. The study was approved by our Institutional Review Board.

### A. Check of diffusion gradients

To check the calibration of diffusion gradient coils, diffusion‐weighted images in the axial plane of a spherical (diameter 17 cm) water phantom at room temperature (21°C) were obtained using a DWI‐SE‐EPI sequence (TR 8000 ms, TE minimum, FOV 24cm×24cm, slice thickness 5 mm, interslice gap 1 mm, number of slices 5, matrix 128×128, number of signals acquired 2). The *b‐value* ranged from 0 to $1750\;\text{sec}/{\text{mm}}^{2}$ with $250\;\text{sec}/{\text{mm}}^{2}$ steps. The *b‐value* was modulated by changing the effective diffusion time while the diffusion gradient strength was fixed. The combinations of the TE and diffusion times $\left(\text{b‐value}(\text{sec}/{\text{mm}}^{2})/\text{TE}(\text{ms})/\Delta (\text{ms})/\delta (\text{ms})\right)$ were: (250/51.5/17.5/13.1), (500/59/21.4/16.8), (750/64.4/24.1/19.5), (1000/68.6/26.2/21.6), (1250/72.2/28/23.4), (1500/75.4/29.6/25), and (1750/78.2/31/26.4). DWI acquisitions were performed applying diffusion encoding gradients pulses independently along the main orthogonal directions: superior/inferior (S/I), right/left (R/L) and anterior/posterior (A/P). The signal S(b) (mean± standard deviation) at different *b‐value* of a circle ROI (7000 mm^2^) in the center of the median slice was measured and the logarithm of signal loss $\left(\ln[S(b)/S_{0}]\right)$ with respect to the unattenuated signal ${(S}_{0})$ was calculated. A linear regression $\left(y=a_{2}-a_{1}x\right)$ of $\left(\ln[S(b)/S_{0}]\right)$ as a function of the applied *b‐value* was carried out. The diffusion coefficient along the main directions and any offset in the application of the *b‐value* were obtained by calculating $a_{1}$ and $a_{2}$ respectively.

### B. DTI measurements

Human brain acquisitions were performed by using DTI schemes with different numbers of diffusion weighting directions. Since DTI measurements could depend on the specific set of directions that is employed, we used optimized DTI acquisition schemes.^(^ ^20^ ^,^ ^30^ ^)^ Accordingly, for each DTI acquisition scheme the diffusion gradients orientations were defined by an electrostatic repulsion algorithm, proposed by Jones et al.,^(28)^ which arranges the gradients vectors uniformly in the space (Table 1. In addition to the scheme with six gradient sampling orientations, the MR scanner can acquire DTI data using acquisition schemes with odd numbers of diffusion‐weighting directions, up to 55. Thus, the combinations of the DTI acquisition schemes (number of directions/number of repeat of each direction) were 6/8, 11/5, 19/3, 27/2, and 55/1, so that the acquisition times were kept similar.

**Table 1:** The x, y and z components of unit vectors that define the diffusion‐weighting directions for the optimized DTI acquisition schemes (N=6,11,19,27,55) based on an electrostatic repulsion algorithm.

*N*	*Encoding Vectors* $\{[g_{x}\;g_{y}\;g_{z}]\}$
6	{[1.000 0.000 0.000], [0.446 0.895 0.000], [0.447 0.275 0.851], [0.448−0.723−0.525],[0.447−0.7240.526],[−0.449−0.2770.850]}
11	{[1.000 0.000 0.000], [0.723 0.691 0.000], [0.069−0.2630.962], [0.725−0.595−0.347], [−0.216−0.8220.528], [−0.694−0.1940.693], [0.477 0.546 0.689], [−0.0200.9690.245], [0.737−0.1380.662], [0.517−0.7770.359], [0.233−0.429−0.872]}
19	{[1.000 0.000 0.000], [0.439 0.898 0.000], [−0.2910.4680.834], [0.848−0.5300.028], [−0.178−0.8690.462], [−0.557−0.2910.778], [0.827 0.506 0.246], [−0.2070.978−0.003], [0.844−0.0370.535], [0.501−0.7580.418], [0.019−0.863−0.505], [−0.386−0.183−0.904], [−0.042−0.5320.846], [−0.5810.7080.401], [−0.4810.375−0.793], [−0.796−0.5040.336], [0.816−0.156−0.556], [0.080−0.046−0.996], [−0.447−0.643−0.621]}
27	{[1.000 0.000 0.000], [0.213 0.977 0.000], [−0.1970.5890.784], [0.870−0.453−0.195], [−0.294−0.7950.531], [−0.833−0.3970.384], [0.632 0.407 0.660], [−0.2330.9630.132], [0.877 0.103 0.470], [0.625−0.780−0.021], [−0.036−0.865−0.501], [0.558−0.7090.432], [−0.523−0.4040.751], [−0.5550.6460.524], [−0.1670.020−0.986], [−0.868−0.481−0.126], [0.604−0.146−0.783], [0.214−0.139−0.967], [−0.613−0.7750.155], [−0.3140.547−0.776], [0.518 0.769 0.374], [−0.5980.151−0.787], [0.870−0.3630.333], [0.240 0.477 0.845], [0.0970.458−0.884], [0.141−0.9020.409], [0.883−0.084−0.462]}
55	{[1.000 0.000 0.000], [0.377 0.926 0.000], [−0.1330.5160.846], [0.907−0.3900.161], [−0.111−0.7970.594], [−0.789−0.3360.514], [0.676 0.266 0.687], [−0.2570.9630.081], [0.829−0.0020.559], [−0.062−0.998−0.019], [0.089−0.929−0.359], [0.414−0.802−0.431], [−0.351−0.5870.730], [−0.6780.6800.278], [−0.5180.003−0.855], [−0.776−0.615−0.142], [0.698−0.204−0.687], [0.420−0.328−0.847], [−0.226−0.901−0.370], [−0.6920.698−0.187], [0.508 0.800 0.320], [−0.6430.234−0.729], [0.954−0.1070.279], [0.162 0.413 0.896], [−0.1110.132−0.985], [0.251−0.9240.287], [0.887−0.440−0.138], [−0.0960.1990.975], [0.0860.946−0.312], [0.943 0.328 0.053], [−0.2860.385−0.878], [−0.3810.0010.925], [−0.269−0.122−0.955], [−0.503−0.2960.812], [−0.944−0.1700.283], [−0.9210.1450.362], [0.493−0.7490.443], [−0.5370.8430.030], [0.879 0.286 0.382], [−0.725−0.0910.683], [0.8220.520−0.233], [0.221−0.7340.642], [−0.4970.5740.651], [−0.1210.7570.643], [0.792−0.4070.455], [−0.174−0.2810.944], [−0.417−0.8310.368], [0.768−0.434−0.472], [0.541−0.5100.669], [0.0210.555−0.832], [−0.643−0.7570.117], [0.6030.609−0.515], [0.673 0.571 0.469], [−0.238−0.714−0.659], [0.452 0.486 0.748]}

Diffusion‐weighted images in the axial plane were obtained with a DWI‐SE‐EPI sequence (TR 8000 ms, TE 79 ms, FOV 24cm×24cm, slice thickness 5 mm, interslice gap 1 mm, number of slices 8, matrix 128×128, *b‐value* $1000\;\text{sec}/{\text{mm}}^{2}$). The images were zero‐filled to 256×256 pixels in‐plane by the MR scanner reconstruction software. The scan time to acquire the entire DTI dataset was approximately 40 minutes.

To reduce head motions between the repeated measurements, the subject's head was secured in the head coil by means of foam padding and a restraining strap stretched across the forehead. All examinations were performed on the same day, thus avoiding any long‐term change of the MR scanner performances. Data from each subject were acquired in a single session so as to avoid the variability associated with repositioning. In each subject session, the order of DTI schemes with different N during acquisitions was randomized to reduce potential bias in the data. The acquired slices included the human brain structures from the cervico‐bulbar junction to the centrum semiovale.

For each DTI scheme, the signal‐to‐noise ratio (SNR) of the acquired images has been measured. Since the SNR in a selected anatomical region in diffusion‐weighted images can depend on the direction of the applied diffusion gradient, the SNR has been evaluated using the diffusion unweighted ${(b}_{0})$ images. In particular, SNR has been calculated as the signal mean value of a ROI placed in the splenium divided by the signal standard deviation of a ROI in the background.

The raw diffusion tensor data were processed with a computer software program (FuncTool; GE Medical Systems); images were corrected for motion artifact and eddy currents distortion. Then, diffusion tensor was estimated from the raw data by using the linear least squares (LLS) method and DTI maps of FA and MD were computed voxelwise:^(^ ^4^ ^,^ ^31^ ^)^

(1) $$FA=\sqrt{\frac{1}{2}}\frac{\sqrt{{\left(\lambda_{1}-\lambda_{2}\right)}^{2}+{\left(\lambda_{2}-\lambda_{3}\right)}^{2}+{\left(\lambda_{3}-\lambda_{1}\right)}^{2}}}{\sqrt{\lambda_{1}^{2}+\lambda_{2}^{2}+\lambda_{3}^{2}}}$$

(2) $$MD=\frac{1}{3}(\lambda_{1}+\lambda_{2}+\lambda_{3})$$

where $\lambda_{1},\lambda_{2}$ and $\lambda_{3}$ are (respectively) the largest, medium and smallest eigenvalue of diffusion tensor. The maps were visually inspected by a single neuroradiologist who had 13 years of experience in interpreting MR imaging.

**Figure 1:**
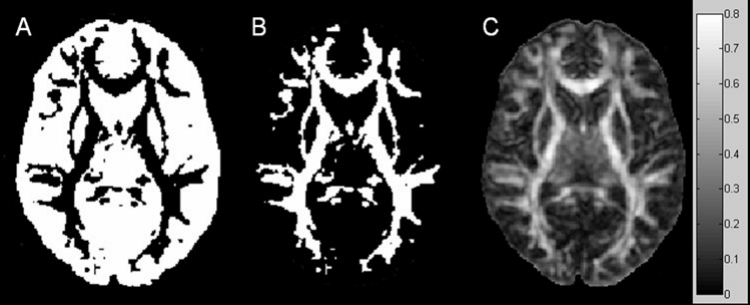
Masks of low (A) and high (B) anisotropy regions and FA map (C) of a healthy subject.

Postprocessing of FA and MD maps was performed by using custom scripts software in MATLAB 6.5 (Mathworks, Natick, MA) running on a PC. For each single subject, masks of low (L, 0≤FA≤0.3) and high (H, 0.3<FA) anisotropy regions were generated (Fig. 1) using a FA threshold. Since in fiber tracking reconstructions a FA value usually in the range 0.15–0.3 is employed as a criterion of termination,^(32)^ a conservative FA threshold of 0.3 was employed in order to separate the main white matter from the surrounding parenchyma. Moreover, in previous studies,^(^ ^33^ ^,^ ^34^ ^)^ gray/white matter segmentation of the brain was performed by using a FA threshold of 0.2–0.3. Thus, we can assume that voxels with FA values above the applied threshold were likely to represent the main white matter, whereas voxels with FA values below the threshold were likely to be the surrounding region. The masks were obtained by using the DTI data set with N=6 and they were applied to segment all maps of DTI derived parameters with different N. The mean value $\left({\text{FA}}_{L},\;{\text{FA}}_{H},\;{\text{MD}}_{L},\;{\text{MD}}_{H}\right)$ and standard deviation $\left(\sigma_{\text{FA}‐L},\;\sigma_{\text{FA}‐H},\;\sigma_{\text{MD}‐L},\;\sigma_{\text{MD}‐H}\right)$ of FA and MD were calculated for each segmented region. With the aim to quantify the capability of FA and MD maps to characterize different brain structures, the contrast‐to‐signal variance ratio (CVR) between the main white matter and the surrounding cerebral region was calculated as follows:^(^ ^3^ ^,^ ^11^ ^)^

(3) $$CVR_{FA}=\frac{FA_{H}-FA_{L}}{\sqrt{\sigma_{FA-H}^{2}+\sigma_{FA-L}^{2}}}$$

(4) $$CVR_{MD}=\frac{MD_{H}-MD_{L}}{\sqrt{\sigma_{MD-H}^{2}+\sigma_{MD-L}^{2}}}$$

The FA and MD data $\left({\text{FA}}_{L},\;{\text{FA}}_{H},\;{\text{CVR}}_{\text{FA}},\;{\text{MD}}_{L},\;{\text{MD}}_{H},\;{\text{CVR}}_{\text{MD}}\right)$ were separately analyzed in a one‐way repeated measures analysis of variance (ANOVA) by means of nonparametric Friedman's test. Then, neighboring data points which were significantly different were assessed by using the statistical Wilcoxon matched pairs test. Correlations between the FA and MD data values and N were investigated by using Pearson (p) and Spearman (s) rank correlation testing.

The DTI measurements were also performed by acquiring an isotropic water phantom. In particular, anisotropy and diffusion maps of a spherical water phantom with a diameter of 17 cm were obtained by employing DTI schemes with N=6, 11, 19, 27, and 55. For each N, the mean of FA and MD values of a circle ROI (7000 mm^2^) in the center of the phantom was measured.

Then, in order to investigate any relevant effect due to the orientation of diffusion‐weighting gradients, the DTI measurements were repeated in another group of six healthy subjects (four females, two males; men age35±4years) by changing the orientation of diffusion weighting gradients (Table 2.

**Table 2:** The x, y and z components of unit vectors that define the diffusion‐weighting directions employed for the repeated DTI measurements by changing the orientations of diffusion weighting gradients.

*N*	*Encoding vectors* $\{[g_{x}\;g_{y}\;g_{z}]\}$
6	{[0.707 0.000 0.707], [−0.7070.0000.707], [0.000 0.707 0.707], [0.0000.707−0.707], [0.707 0.707 0.000], [−0.7070.7070.000]}
11	{[0.153−0.8310.534], [0.0930.692−0.716], [0.686 0.725 0.060], [0.101 0.258 0.961], [−0.143−0.243−0.959], [−0.9100.201−0.363], [−0.4050.8730.272], [0.897−0.1850.402], [0.731−0.415−0.541], [−0.440−0.865−0.242], [−0.774−0.2040.600]}
19	{[−0.4940.1500.856], [0.161−0.2510.954], [0.217 0.651 0.728], [0.515−0.793−0.327], [−0.313−0.942−0.116], [0.028 0.999 0.016], [0.785 0.079 0.614], [−0.502−0.6540.566], [−0.029−0.561−0.827], [−0.791−0.362−0.493], [0.6320.016−0.775], [0.402−0.7980.448], [0.7850.618−0.044], [−0.6260.7050.333], [−0.979−0.0740.189], [0.2170.710−0.670], [−0.2720.164−0.948], [0.975−0.211−0.072], [−0.7110.555−0.431]}
27	{[−0.8300.5480.106], [0.092−0.0540.994], [−0.2630.9240.279], [−0.495−0.8110.313], [−0.4620.764−0.451], [−0.3840.247−0.890], [0.789−0.062−0.611], [0.212−0.146−0.966], [0.669 0.221 0.710], [0.988 0.098 0.118], [0.065−0.6950.716], [−0.5650.3780.733], [0.2490.534−0.808], [−0.530−0.3020.792], [0.686−0.4480.573], [−0.398−0.447−0.801], [0.7600.560−0.330], [0.300−0.9460.125], [0.1640.953−0.254], [−0.813−0.529−0.244], [−0.951−0.1200.286], [0.074 0.614 0.786], [0.552 0.777 0.302], [−0.217−0.922−0.320], [0.830−0.548−0.106], [0.364−0.707−0.607], [−0.8870.119−0.446]}
55	{[−0.388−0.2960.873], [−0.946−0.0810.313], [0.923−0.3850.029], [−0.445−0.161−0.881], [0.089−0.9300.357], [−0.3990.8290.393], [−0.7070.0320.706], [−0.4420.343−0.829], [0.9850.071−0.160], [−0.8170.4490.362], [−0.973−0.160−0.165], [0.3660.925−0.098], [−0.698−0.4750.536], [−0.1940.980−0.047], [0.422−0.627−0.655], [0.918−0.0590.392], [−0.9510.302−0.061], [0.482−0.174−0.859], [−0.0180.566−0.824], [−0.008−0.843−0.537], [−0.0220.064−0.998], [0.303−0.6400.706], [0.606 0.751 0.263], [−0.8010.040−0.597], [−0.198−0.6940.692], [−0.822−0.5650.066], [−0.532−0.807−0.257], [0.900 0.383 0.207], [0.044−0.417−0.908], [−0.6540.756−0.023], [0.380 0.645 0.662], [0.819−0.267−0.508], [0.721−0.4810.499], [0.549−0.8060.222], [0.696−0.670−0.258], [−0.365−0.606−0.707], [0.091−0.2730.958], [−0.5120.4710.719], [0.693 0.289 0.660], [−0.7520.486−0.446], [0.7810.586−0.217], [0.101 0.933 0.347], [−0.2200.1580.963], [0.7800.224−0.584], [−0.755−0.439−0.487], [0.4790.666−0.573], [−0.153−0.986−0.059], [0.3890.293−0.874], [0.0670.886−0.459], [0.307−0.935−0.179], [−0.0720.6410.764], [−0.457−0.8390.296], [−0.3920.776−0.493], [0.550−0.1600.819], [0.253 0.233 0.939]}

## III. RESULTS

### A. Check of diffusion gradients

**Figure 2:**
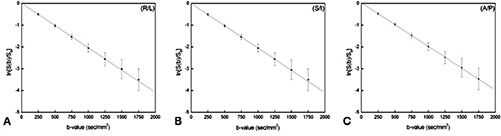
The logarithm of water phantom signal loss $\left(\text{ln}[S(b)/S_{0}]\right)$ as a function of *b‐value*. The diffusion sensitizing gradient pulse was applied along the R/L (A), S/I (B) and A/P (C) directions.

The linear regression described very well the experimental data (Fig. 2). The linear correlation coefficient (r) and the p‐value (*p*) were (respectively) less than −0.99 and $0.0001\;(r_{R/L}=-0.99,\;p_{R/L}<0.0001;r_{S/I}=-0.99,\;p_{S/I}<0.0001;\;r_{A/P}=-0.99,\;p_{A/P}<0.0001$) for each main direction (R/L, S/I, A/P). The diffusion coefficient values $\left(a_{1}\right)$ were $\left(2.04\pm 0.14)\times 10^{-3}\;{\text{mm}}^{2}/\text{sec}\;(R/L),\;(2.00\pm 0.14)\times 10^{-3}\;{\text{mm}}^{2}/\text{sec}\;(A/P\right)$, and $\left(2.04\pm 0.14)\times 10^{-3}\;{\text{mm}}^{2}/\text{sec}\;(S/I\right)$; while $a_{2}(R/L)=(-0.002\pm 0.076),\;a_{2}(S/I)=(-0.005\pm 0.071)$, and $a_{2}(A/P)=(0.026\pm 0.074)$.

### B. DTI measurements

**Figure 3:**
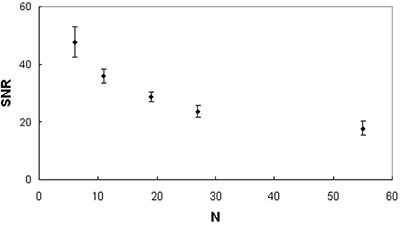
Signal‐to‐noise ratio data of diffusion unweighted images ${(b}_{0})$ of a healthy subjects group: SNR values (mean±standard deviation) as a function of N.

**Figure 4:**
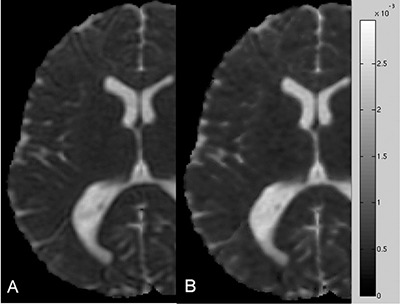
MD (mm^2^/sec) maps of the same healthy subject obtained by using DTI acquisition schemes with N=6 (A) and N=55 (B) do not reveal appreciable differences in detecting brain structures.

**Figure 5:**
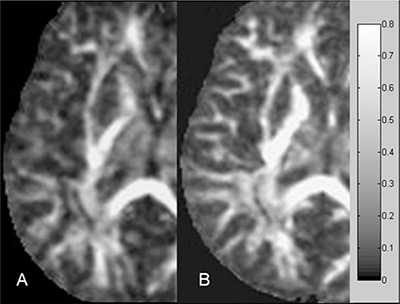
FA maps of the same healthy subject obtained by using DTI acquisition schemes with N=6 (A) and N=55 (B). The increased contrast between gray and white matter in the image (B) with respect to image A allows a better delineation of the gray‐white matter junction that is recognizable along all the white matter borders. In particular, the improvement of image quality is detectable in the insular circumvolutions where the subcortical “U” fibers are clearly visualized only on image (B).

**Figure 6:**
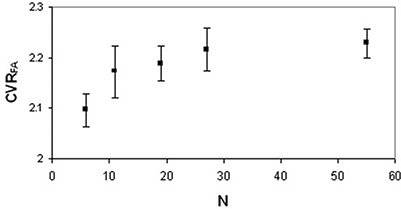
Contrast‐to‐signal variance ratio data of human brain FA maps of a healthy subjects group: ${\text{CVR}}_{\text{FA}}$ values (mean±standard error of the mean) as a function of N.

DWI images were not corrupted by artifacts and the signal‐to‐noise ratio of acquired images decreased as N increased (Fig. 3). Upon qualitative visual inspection, MD maps obtained by employing DTI acquisition schemes with different N did not reveal appreciable differences in detecting brain structures (Fig. 4). On the other hand, FA maps showed both an increased contrast between the white matter and the surrounding gray matter and a better delineation of white matter fiber bundles as the number of diffusion‐weighting directions increased (Fig. 5). Analysis of variance indicated that ${\text{CVR}}_{\text{FA}}$ values significantly (p<0.016) depend on N, whereas ${\text{CVR}}_{\text{MD}}$ values do not (p>0.05). In ${\text{CVR}}_{\text{FA}}$ analysis, the neighboring data points that were significantly different (p=0.046) were N=6 and N=11. The ${\text{CVR}}_{\text{FA}}$ mean values significantly $\left(r_{s}=1,\;p_{s}<0.05\right)$ increased (~6.5%,0.13) as the number of diffusion weighting directions increased (Fig. 6).

**Figure 7:**
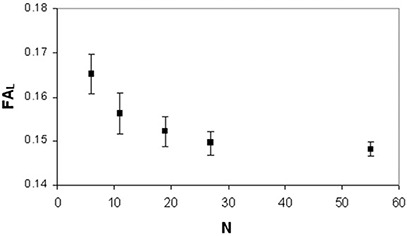
Human brain anisotropy data of a healthy subjects group: ${\text{FA}}_{L}$ values (mean±standard error of the mean) as a function of N.

**Figure 8:**
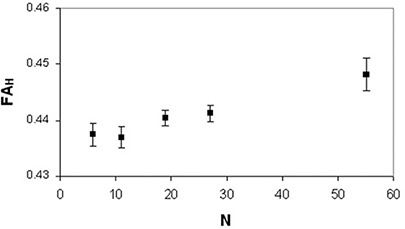
Human brain anisotropy data of a healthy subjects group: ${\text{FA}}_{H}$ values (mean±standard error of the mean) as a function of N.

**Figure 9:**
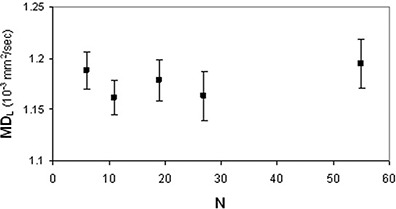
Human brain diffusion data of a healthy subjects group: ${\text{MD}}_{L}$ values (mean±standard error of the mean) as a function of N.

**Figure 10:**
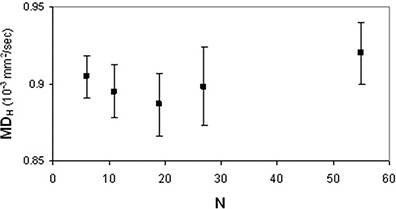
Human brain diffusion data of a healthy subjects group: ${\text{MD}}_{H}$ values (mean±standard error of the mean) as a function of N.

Analysis of variance of both ${\text{FA}}_{L}$ (p<0.005) and ${\text{FA}}_{H}(p<0.03)$ data showed significant differences among the DTI acquisition schemes with different N. In FAL analysis, the neighboring data points which were significantly different (p=0.028) were N=6 and N=19. As for ${\text{FA}}_{H}$ analysis, the neighboring data points which resulted significantly different (p=0.028) were N=19 and N=55. ${\text{FA}}_{L}$ values (Fig. 7) significantly $\left(r_{s}=-1,\;p_{s}<0.05\right)$ decreased (~11%,0.017) as N increased, whereas ${\text{FA}}_{H}$ values (Fig. 8) significantly $\left(r_{p}=0.98,\;p_{p}=0.002\right)$ increased (~2.5%,0.011) as N increased. The analysis of variance showed no significant (p>0.05) dependence of ${\text{MD}}_{L}$ (Fig. 9) and ${\text{MD}}_{H}$ (Fig. 10) on the number of diffusion weighting directions.

**Figure 11:**
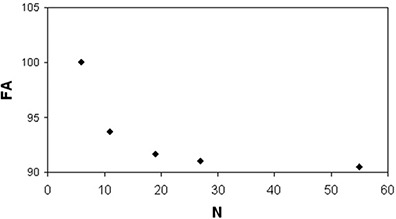
FA values of an isotropic water phantom obtained by applying DTI acquisition schemes with different N. The anisotropy values are normalized (100) to the FA value (0.054) measured by using the DTI scheme with N=6.

The FA values of the isotropic water phantom (Fig. 11) decreased (~10%) as the number of diffusion weighting directions increased, whereas MD values showed random variations.

As for the repeated human brain DTI measurements performed with varying the diffusion‐weighting directions, analysis of variance confirmed that ${\text{FA}}_{L}\;(p<0.007),\;{\text{FA}}_{H}\;(p<0.02)$ and ${\text{CVR}}_{\text{FA}}\;(p<0.006)$ significantly depend on N, whereas ${\text{MD}}_{L},\;{\text{MD}}_{H}$ and ${\text{CVR}}_{\text{MD}}$ do not (p>0.05). ${\text{FA}}_{L}$ values significantly $\left(r_{s}=-1,\;p_{s}<0.05\right)$ decreased (~6%,0.010) as N increased, whereas ${\text{FA}}_{H}$ values significantly $\left(r_{p}=0.95,\;p_{p}=0.015\right)$ increased (~1.6%,0.008) as N increased. ${\text{CVR}}_{\text{FA}}$ significantly $\left(r_{s}=0.9,\;p_{s}<0.037\right)$ increased (~4%,0.08) as N increased.

## IV. DISCUSSION

The noise can couple into anisotropy measurements affecting the rotational variance of FA values.^(^ ^22^ ^–^ ^24^ ^)^ Moreover, it has been suggested that increasing N confers the least sensitivity to rotational variance due to noise.^(^ ^17^ ^–^ ^21^ ^)^ Pierpaoli and Basser^(31)^ have shown by means of Monte Carlo simulation that noise affects also the accuracy of FA values. In particular, FA values are generally overestimated due to noise. This effect increases with the noise and it is greater for isotropic structures rather than for anisotropic structures. The FA overestimation has been shown to be related to an overestimation and underestimation respectively of $\lambda_{1}$ and $\lambda_{3}$. Since the effect of noise on rotational variance of anisotropy measurements has proved to be biased by N, we hypothesized that also the accuracy of FA and MD values can be dependent on N. Ni et al.^(26)^ have previously investigated this effect only in strongly directional white matter, at ROI and voxel level, by using a limited number of DTI acquisition protocols (N=6, 21, 31). Both FA and MD have shown no significant differences at ROI level. However, at the voxel level the FA correlation coefficient $r_{21-31}$ between the DTI acquisition schemes with N=21 and N=31 has been shown to be higher than $r_{6-21}$ and $r_{6-31}$. However, no upstream or downstream trend with N has been reported. The MD correlation coefficients between the DTI acquisition schemes with different N have displayed random variations. In this regard, also the single‐subject study of Landman et al.^(27)^ about the reproducibility of DTI measurements seems to indicate that FA can be more consistently biased with respect to MD. In the present study, we performed human brain measurements of FA and MD in both high and low anisotropy segmented brain regions by employing different N up to 55. Furthermore, the contrast‐to‐signal variance ratio between different brain regions in FA and MD maps was calculated.

Diffusion‐weighted images are obtained by using diffusion encoding gradient pulses produced by three independent gradient coils whose well functioning is fundamental for a reliable quantification of diffusion parameters. Therefore, before carrying out human brain DTI measurements, diffusion gradient coils calibration was checked by performing water phantom measurements. The logarithm of the measured water phantom signal loss linearly decreased as the applied *b‐value* increased within a typical range of interest in clinical practice $\left(0-1750\;\text{sec}/{\text{mm}}^{2}\right)$. The measured diffusion coefficient values ${(a}_{1})$ of the water phantom along the main orthogonal directions were in agreement with the water diffusion coefficient at room temperature.^(35)^ Moreover, the measured $a_{2}$ values indicated no substantial offset in the application of the *b‐value*.

The ANOVA of the human brain anisotropy data obtained by means of different DTI acquisition schemes indicated that FA values significantly vary as N increases. It is noteworthy that we revealed a decrease (~11%, 0.017) of FA values in regions of low anisotropy $\left({\text{FA}}_{L}\right)$ and an increase (~2.5%, 0.011) of FA values in regions of high anisotropy $\left({\text{FA}}_{H}\right)$. The decrease of ${\text{FA}}_{L}$ values was in agreement with the variation of FA values of the isotropic water phantom. Since studies of FA rotational variance have indicated that the effect of noise on anisotropy measurements is reduced when N increases, the anisotropy overestimation due to noise is reduced also and this can explain the decrease of ${\text{FA}}_{L}$ values when N increases. The effect of noise is lesser in high anisotropic structures rather than in isotropic structures. Nonetheless, high anisotropic white matter fibers are likely to be aligned along one of the diffusion gradients when acquisition schemes with high N, sampling the space more uniformly, and reducing any directional bias are employed. Thus, in high anisotropy regions this may allow a more accurate measurement of diffusion both along and orthogonally to white matter fiber bundles, resulting in a slight increase of ${\text{FA}}_{H}$ values when N increases. Notwithstanding ${\text{FA}}_{L}$ decreased with N, we observed that it sharply changed from N=6 to N=19, whereas it changed little from N=19 to N=55. This is consistent with the Monte Carlo studies of Papadakis et al.^(17)^ and Jones^(21)^ where it has been suggested that at least 20 sampling orientations seem to be necessary for a robust estimation of anisotropy in terms of diffusion tensor orientation independence.

Upon visual inspection, FA maps seemed to be characterized by an improved contrast between different cerebral structures as N increased (Fig. 5). This corroborated the qualitative observations of Skare et al.^(20)^ and Jones et al.^(28)^ Moreover, the quantitative analysis indicated that ${\text{CVR}}_{\text{FA}}$ mean value significantly increased (~6.5%) with the number of diffusion‐weighting directions allowing a better visualization of white matter. This result demonstrates that the capability of FA maps to differentiate brain structures can be improved as N increases.

The ${\text{MD}}_{L},\;{\text{MD}}_{H}$ and ${\text{CVR}}_{\text{MD}}$ values did not significantly vary with N. The human brain MD results were in agreement with the water phantom MD results. Unlike FA measurements, MD measurements do not seem to depend on the number of diffusion‐weighting directions both in low and high anisotropic regions. It should be noted that FA and MD are two DTI derived parameters which characterize respectively anisotropy and diffusivity. Therefore, FA and MD have a different geometrical interpretation: FA measures the shape of the diffusion ellipsoid, whereas MD measures the size of the diffusion ellipsoid.^(4)^ Based also on the study of Pierpaoli and Basser,^(31)^ the FA changes that we revealed can be ascribed to an opposite variation of $\lambda_{1}$ and $\lambda_{3}$ – which is likely to not imply a significant difference of MD values.

In this study, the diffusion tensor (D) was estimated from the raw data by using the linear least squares (LLS) method. The LLS method is mostly widely used, although the nonlinear least squares method (NLS) can be applied as well. Simulations studies have suggested that the NLS method seems to show a lower mean squared error in estimating DTI derived parameters.^(^ ^36^ ^,^ ^37^ ^)^ Nonetheless, for both methods the SNR has proved to be the main limiting factor in terms of reliability of FA measurements.^(37)^ In this regard, the present study indicates that the effect of noise on maps of FA can be reduced by increasing the number of diffusion‐weighting directions.

We suppose that our results are independent of the orientation of the DTI acquisition schemes. To investigate this effect, the DTI measurements were repeated in a group of six healthy subjects by changing the orientation of diffusion‐weighting gradients. The additional measurements confirmed our main findings, showing that ${\text{FA}}_{L}$ significantly decreases as N increases whereas ${\text{FA}}_{H}$ and ${\text{CVR}}_{\text{FA}}$ significantly increase as N increases.

We recognize as a limit of our study that DTI acquisitions were performed on a single MR scanner system. In this regard, more exhaustive studies may be needed to obtain a generalization of our conclusions. However, our results indicate that, in clinical practice, FA values can significantly vary using DTI schemes with different numbers of diffusion‐weighting directions. Therefore, before beginning a clinical study of anisotropy it is suggested to assess for each MR scanner the effect of employing different N. Nonetheless, we are inclined to suppose that our results do not represent only a characterization of a specific MR scanner system but that they could indicate a more general effect of N on DTI measurement of human brain for the following reasons: a) the well functioning of the MR scanner diffusion gradients was assessed by performing a preliminary water phantom verification; b) in regions of low anisotropy, FA decreased as N increased whereas, in regions of high anisotropy, FA increased as N increased. We speculate that the opposite variation of FA values in low and high anisotropy regions when DTI schemes with different N are applied is likely to exclude a systematic bias due to the specific performances of our MR scanner. Indeed, any effect of N due only to the specific performance of the MR scanner would have similarly biased FA values of different human brain regions.

## V. CONCLUSIONS

This experimental study complements theoretical analyses concerning the effect of N on rotational variance of diffusion measurements and it provides further insight into the dependence of the accuracy of FA and MD maps on the employed DTI acquisition scheme. No significant difference of human brain MD values on the number of diffusion‐weighting directions was revealed. On the other hand, FA values significantly varied when DTI acquisition schemes with different N were employed. It is noteworthy that FA of high anisotropic structures, such as the main white matter, increased as N increased whereas FA of low anisotropic regions decreased as N increased. We demonstrated that, in FA maps, the contrast‐to‐signal variance ratio between the main white matter and the surrounding regions significantly increases as N increases allowing a better delineation of the gray–white matter junction. In clinical practice, this is likely to allow a better depiction of cortical lesions with respect to cortical lesions extending to subcortical white matter when DTI acquisition schemes with N greater than 20 are employed. Since the FA variation due to N is comparable to the anisotropy change revealed in some pathological diseases^(^ ^7^ ^,^ ^9^ ^,^ ^11^ ^,^ ^38^ ^)^ (5%–15%), in clinical studies the effect of N may represent a confounding variable for anisotropy measurements. Therefore, it is recommended that group comparison studies and longitudinal studies should be performed by using the same DTI scheme with fixed N for all subjects’ acquisitions. Moreover, any dependence of FA values on N should be taken into account when fiber tracking reconstructions are performed. Indeed, several algorithms which reconstruct a fiber bundle's pathway beginning from a certain point of origin employ a FA value as a criterion of termination.^(32)^

## ACKNOWLEDGEMENTS

The authors would like to thank Piero Ghedin (GE Healthcare) for expert technical assistance.
